# Beyond Ocular Toxicity: Cerebrovascular Events After Intra-Arterial Chemotherapy for Retinoblastoma

**DOI:** 10.3390/jcm15124829

**Published:** 2026-06-22

**Authors:** Yacoub A. Yousef, Alaa Tarazi, Mona Mohammad, Hadeel Halalsheh, Qusai F. Abu Salim, Dima Abu Laban, Reem AlJabari, Mustafa Mehyar, Hazem Haboob, Ibrahim AlNawaiseh

**Affiliations:** 1 Department of Surgery (Ophthalmology), King Hussein Cancer Centre (KHCC), Amman 11941, Jordan; alaatarazi11@gmail.com (A.T.); dr.qusaiabusalim@gmail.com (Q.F.A.S.);; 2 Department of Pediatrics Oncology, King Hussein Cancer Centre (KHCC), Amman 11941, Jordan; hadeelhalalsheh@khcc.jo; 3 Department of Radiology, King Hussein Cancer Centre (KHCC), Amman 11941, Jordan

**Keywords:** intra-arterial chemotherapy, retinoblastoma, cerebrovascular accidents, stroke

## Abstract

**Background**: Cerebrovascular accidents (CVAs) are among the most serious complications of intra-arterial chemotherapy (IAC) for retinoblastoma (RB). This study evaluated the incidence and potential risk factors of this rare event. **Methods**: A retrospective cohort study included RB patients who received IAC at a tertiary cancer center. Diagnosis of CVAs was based on clinical and/or neuroimaging findings. Data included demographics, tumor features, complications, and outcomes. Meta-analysis was not feasible due to heterogeneity. A systematic review following PRISMA guidelines was conducted across major databases up to December 2025, including studies reporting CVA after IAC. **Results**: The cohort included 33 children who underwent 104 IAC procedures (Melphalan). CVA occurred in three patients (3/33 (9%) of patients, and 3/104 (2.9%) of procedures). Two were confirmed by neuroimaging, while one was a transient ischemic attack. Two patients (67%) were girls, and 2 of 3 (67%) were younger than 1 year. All events occurred during the IAC procedure and were ipsilateral to the treated eye. Two patients had no residual neurological deficits, while one showed improvement with only a minor residual deficit. The systematic review included 14 studies with 932 patients and identified 11 CVA events (1.2%; Range 0–9.1% per patient and 0–2.2% per IAC procedure). All were ischemic with variable presentations. Younger age, repeated catheterization, vasospasm, and embolic events were common risk factors. Outcomes were generally favorable. **Conclusions**: CVA after IAC, though rare, may be underreported. Events are likely procedure-related and influenced by age, treatment intensity, and vascular toxicity. Careful technique, close monitoring, and standardized reporting are needed to recognize/reduce the real risk.

## 1. Introduction

Retinoblastoma (RB) is the most common primary intraocular malignancy in the pediatric population (1/15,000), accounting for approximately 3% of all childhood cancers [1,2]. Over the past decade, treatment strategies have evolved significantly, with a major emphasis on globe preservation and avoidance of enucleation whenever possible. Management options include systemic intravenous chemotherapy (IVC), focal therapies such as laser photocoagulation, cryotherapy, and transpupillary thermotherapy (TTT), intra-arterial chemotherapy (IAC), intravitreal chemotherapy (IViC), and, in selected cases, external beam radiotherapy (EBRT) [3,4,5].

Over the past decade, IAC has become an established treatment modality for RB. The technique was first introduced by Suzuki and Kaneko [6] and was later revisited by Abramson et al. in 2008 [7], building on the super selective catheterization method for the brain described by Gobin et al. in 2001 [8]. IAC involves super selective catheterization of the ophthalmic artery under fluoroscopic guidance, followed by direct injection of chemotherapy into the eye globe to achieve high intraocular drug concentrations while minimizing systemic toxicity.

Multiple studies have evaluated the safety and efficacy of IAC for RB [9,10,11,12]; however, awareness of rare but potentially serious complications during or shortly after treatment remains essential. One such complication is cerebrovascular accidents (CVA), including stroke, hemorrhage, and transient ischemic attack (TIA), which may arise from thromboembolism, arterial spasm, or vessel injury during super selective catheterization of the ophthalmic artery [13]. While most reported events are minor or transient, isolated cases of severe stroke have been described. To date, no studies have systematically assessed the prevalence or risk factors of CVA in this pediatric population.

Our study aimed to evaluate the incidence and potential risk factors for CVA as extraocular vascular events in a cohort of RB patients treated by IAC, alongside a systematic review of previously reported CVA cases in the literature.

## 2. Methods and Materials

### 2.1. Case Series

#### 2.1.1. Study Design and Patient Selection

This is a retrospective study of 33 eyes from 33 patients with a clinical diagnosis of intraocular RB who were treated with IAC (melphalan). The study period spans from January 2015 to December 2025. Each patient underwent a comprehensive ophthalmic examination performed under general anesthesia that included fundus photos taken with the RetCam system (Natus Medical Incorporated, Pleasanton, CA, USA) for documentation. Selection required access to patients’ medical, radiological, and pathological reports and fundus Ret-Cam images. Data collected included the patient’s demographics, tumor features, treatment modalities, follow-up, and outcomes. The IAC was offered as a primary or secondary treatment to patients with intraocular RB with an absence of anterior segment invasion, absence of extra-scleral or optic nerve invasion, and older than 6 months of age.

We have included the eyes with the clinical diagnosis of RB that were treated at one stage by IAC and subsequently had clinical and/or radiologic diagnosis of CVA. Patients with pre-existing cerebrovascular disease or incomplete records were excluded.

#### 2.1.2. Data Collection and Definitions

Stroke diagnosis was established based on acute neurological deficits consistent with cerebrovascular insult based on clinical and/or neuroimaging findings (magnetic resonance imaging and/or computed tomography), as interpreted by pediatric neurology and neuroradiology specialists. For each case, detailed data were extracted using a standardized form, including demographic characteristics (age at diagnosis, sex, laterality), tumor classification, IAC-related variables (age at time of IAC session, number of cycles, chemotherapeutic agents administered, catheterization details, and procedural complications), stroke characteristics (time to onset after IAC, clinical presentation, type and vascular territory of stroke, imaging findings, and acute management), and outcomes (neurological sequelae, functional status at last follow-up, tumor control, and survival).

#### 2.1.3. Statistical Analysis and Ethical Considerations

Given the small sample size, the analysis was primarily descriptive. Findings were summarized narratively, and categorical variables were reported as frequencies and percentages. Where appropriate, associations between variables were assessed using Fisher’s exact test. All analyses were conducted using IBM SPSS Statistics version 26 (IBM Corp., Armonk, NY, USA), with a *p*-value < 0.05 considered statistically significant. The study protocol was reviewed and approved by the Institutional Review Board (IRB) at KHCC (IRB No: 25KHCC001, approval date: 20 January 2025). All procedures involving human participants were conducted in accordance with the ethical standards of the institutional research committee and with the principles of the Declaration of Helsinki and its later amendments.

### 2.2. Systematic Review

#### 2.2.1. Search Strategy

A systematic review was conducted in strict adherence to the Preferred Reporting Items for Systematic Reviews and Meta-Analyses (PRISMA) guidelines [14]. The review was prospectively registered with the Prospective Register of Systematic Reviews (PROSPERO) (CRD420251272568). A comprehensive search of the PROSPERO database confirmed the absence of any prior systematic reviews or meta-analyses addressing the same subject matter.

To identify relevant studies, an exhaustive search was performed across four databases: PubMed, Scopus, Web of Science (WOS), and Cochrane, up to 27 December 2025. The search strategy employed the following terms: (retinoblastoma*) AND (“intra-arterial chemotherapy*” OR intraarterial chemotherapy* OR “ophthalmic artery chemotherapy” OR “selective ophthalmic artery infusion” OR “super selective ophthalmic artery*” OR melphalan OR carboplatin OR topotecan) AND (stroke OR “cerebrovascular accident*” OR “cerebrovascular event*” OR “ischemic stroke” OR “hemorrhagic stroke” OR “cerebral infarct*” OR “brain ischemia” OR seizure* OR “neurologic complication*” OR “neurologic adverse event*” OR “vascular complication*” OR vasculopathy). In addition, the reference lists of all included studies and relevant review articles were manually screened to identify any additional eligible studies not captured through the electronic database search.

Studies were considered eligible if they met the following criteria: (1) published in the English language; (2) included pediatric patients diagnosed with RB who underwent intra-arterial chemotherapy (IAC); and (3) reported cerebrovascular events (CVA), including ischemic or hemorrhagic stroke, following IAC, including studies in which no CVA events were observed. No restrictions were applied regarding publication year. Eligible study designs included observational studies (cohort and case–control studies), case series, and case reports. Conference abstracts, review articles, randomized controlled trials, editorials, commentaries, and non-original studies were excluded. Studies that did not involve pediatric RB patients treated with IAC, did not report cerebrovascular outcomes, assessed IAC complications without mentioning CVA, or contained overlapping or duplicate data were excluded.

#### 2.2.2. Screening

The studies were initially screened based on titles and abstracts, followed by a full-text review in accordance with the specified inclusion and exclusion criteria. Two authors independently conducted the search and screening process. Any discrepancies between the reviewers were resolved through discussion with the senior investigator.

#### 2.2.3. Data Collection and Data Items

Data was extracted using a standardized data collection form developed in Microsoft Excel (Office 2019). Two reviewers independently performed data extraction, and the extracted information was cross-verified to ensure accuracy. Any discrepancies were resolved through discussion, with arbitration by a senior author when necessary.

Extracted data included: study characteristics (year of publication, country, study design, study period, total number of patients who underwent IAC, number of IAC sessions, number of CVA events, and duration of follow-up); patient characteristics (age, sex, genetic status (hereditary versus sporadic RB), comorbidities including prothrombotic disorders, cardiac anomalies, vascular abnormalities, or prior CVA, laterality of disease, and International Classification of Retinoblastoma (ICRB) group); RB treatment details (prior therapies, indication for IAC (primary, secondary, or salvage), number of IAC cycles prior to CVA, chemotherapeutic agents used, and dose per cycle); procedural characteristics (vascular access route, catheterization technique, procedure duration, use of anticoagulants, antiplatelet agents or vasodilators, and intra-procedural blood pressure management); CVA-related variables (type of stroke, timing in relation to IAC, clinical presentation, imaging modality, anatomical location, and management); and outcomes (neurological outcome, oncologic outcome including continuation or discontinuation of IAC, switch to alternative therapy, and eye salvage versus enucleation). Reported or proposed risk factors for CVA were also extracted when available.

#### 2.2.4. Risk of Bias Assessment

The quality of each article was evaluated using the Newcastle–Ottawa Scale (NOS) for cohort studies and the Joanna Briggs Institute Critical Checklist for Case Reports (JBI, Adelaide, South Australia) for case reports [15,16]. Two of the reviewers assessed the quality of each included study independently. They compared their results, and disagreements were resolved by detailed discussion.

#### 2.2.5. Data Synthesis

Given the clinical and methodological heterogeneity among included studies, a meta-analysis was not appropriate. Therefore, a narrative synthesis was undertaken in accordance with established guidance for systematic reviews of observational studies. Data were tabulated and summarized at both study and patient levels. Incidence proportions of CVA among patients receiving IAC were calculated when sufficient denominator data were available. Clinical, procedural, and treatment-related characteristics were compared descriptively to identify recurrent patterns and potential risk factors. Outcomes were synthesized qualitatively, focusing on neurological recovery, mortality, and oncologic management decisions following CVA.

## 3. Results

### 3.1. Case Series

This study included 33 eyes from 33 RB patients who received a total of 104 IAC injections by the same interventional neuroradiologist with experience in IAC for RB. The mean age at diagnosis was 19 months, and the mean age at the time of IAC was 24 months. Of the patients, 12 were male (36.4%), and 21 (63.6%) were female. Disease laterality was nearly evenly distributed, with 16 unilateral and 17 bilateral cases, and four (12%) had a positive family history of RB. Most treated eyes (76%) were classified as advanced disease (ICRB groups D or E). IAC was administered as primary treatment in 12 (36.4%) of cases and as secondary treatment in 21 (63.6%), with a median of three injections per patient. CVA occurred in 3 of 33 patients (9.1%) and in 3 of 104 IAC procedures (2.9%) during the treatment course. Among the three cerebrovascular events identified in our institutional cohort, one patient developed an imaging-confirmed ischemic stroke secondary to internal carotid artery dissection, one developed a clinically diagnosed transient ischemic attack without radiologic evidence of infarction, and one experienced MRI-documented watershed hypoxic–ischemic injury associated with severe vasospasm during attempted catheterization. Further cohort characteristics are summarized in Table 1.

Three patients in our cohort developed CVA during IAC: two females and one male. The first female was 9 months old at the time of IAC, the second female was 25 months old, and the male patient was 7 months old. The 9-month-old female and the male patient received IAC as a secondary treatment following prior systemic chemotherapy, whereas the other female received IAC as a primary treatment. The two female patients underwent 2 and 4 injections, respectively, while the male patient received 3 injections. Regarding melphalan dosage, the 9-month-old female and the male patient each received 20 micrograms per injection, whereas the other female received 25 micrograms.

**Figure 1:**
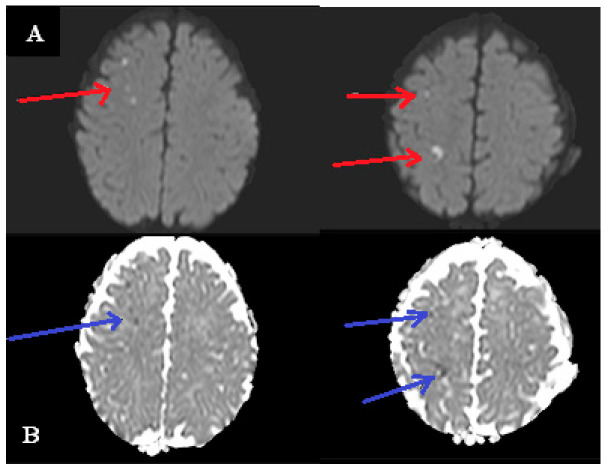
There are small foci of hyperintense signal (red arrows) on DWI (**A**) with corresponding hypointense signal (blue arrows) on the ADC map (**B**) within the white matter of the right cerebral hemisphere, seen at different axial levels. These findings are consistent with areas of restricted diffusion, in keeping with acute infarction.

**Case 1:** A 3-month-old male presented with leukocoria in the right eye and was diagnosed with bilateral RB. Examination under anesthesia revealed total retinal detachment with a large tumor in the right eye (Group D) and multiple smaller tumors in the left eye (Group B). MRI confirmed intraocular disease without intracranial involvement. He received six cycles of systemic chemotherapy (Vincristine/Carboplatin alternating with Vincristine/Topotecan) combined with focal consolidation therapy, followed by IAC (Melphalan) for the right eye at the age of 7 months. The first IAC cycle was successful with a good response. The second and third attempts were aborted due to severe vasospasm of the internal carotid artery despite Nifedipine administration, preventing catheter advancement to the ophthalmic artery. Following the second aborted attempt, MRI showed watershed hypoxic changes (Figure 1), though these were not expected to result in long-term deficits. Given recurrent vasospasm and documented cerebral hypoxia, IAC was discontinued due to high neurological risk. Management was shifted to extensive focal therapy, achieving tumor control without further neurological complications.

**Case 2:** A 3.5-year-old girl with bilateral RB (Group E) was treated with eight cycles of systemic chemotherapy (Vincristine/Carboplatin/Etoposide) combined with focal therapy. Due to advanced disease, the left eye was enucleated. The right eye showed significant tumor regression but had persistent active tumor margins and subretinal seeding. After completing systemic therapy, IAC with Melphalan was initiated for the right eye; the age at first injection was 9 months. The procedure was uneventful; however, the following day, the patient developed an acute focal neurological event characterized by left-sided facial twitching, tonic–clonic movements of the left hand, and left-sided weakness. She was admitted to the ICU and treated with Levetiracetam and Dexamethasone. Neuroimaging showed a small right parietal white matter blooming artifact of uncertain significance, with no evidence of acute infarction or enhancement on MRI. These findings were suggestive of a TIA, based on clinical presentation without corresponding radiological evidence. CT angiography demonstrated patent cerebral vasculature without thrombosis or hemorrhage, and the EEG was normal. The patient recovered fully, remained seizure-free on follow-up, and continued antiepileptic therapy. Subsequent ocular assessments confirmed a good response to IAC, and enucleation was avoided.

**Figure 2:**
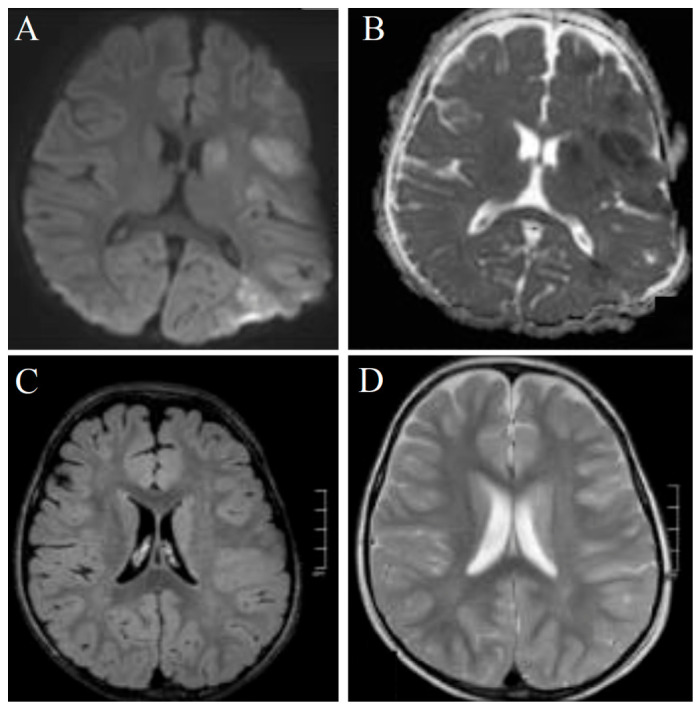
Brain MRI demonstrating a hyperacute ischemic infarction in the left middle cerebral artery (MCA) territory. Diffusion-weighted imaging (DWI) shows a focal hyperintense lesion in the left cerebral hemisphere (**A**) with corresponding hypointensity on the apparent diffusion coefficient (ADC) map (**B**), confirming restricted diffusion. No corresponding signal abnormality is observed on FLAIR (**C**) or T2-weighted imaging (**D**), consistent with a hyperacute stage of infarction before the development of conventional MRI changes.

**Case 3:** A 2-year-old girl presented with a unilateral Group D RB in the left eye, characterized by an amelanotic white mass with extensive vitreous and subretinal seeding. MRI showed no extra-scleral extension, optic nerve involvement, or metastasis. She was treated by primary IAC (Melphalan) at the age of 25 months. The first three cycles were uneventful, with good tumor response. During the fourth cycle, she developed an acute cerebrovascular event due to left ICA occlusion due to dissection. Stroke protocol achieved successful recanalization within 30 min, and she required intubation for 48 h. Post-procedure MRI revealed multiple acute infarctions in the left frontal, parietal, and basal ganglia regions, with microbleeds and signs of carotid dissection or vasospasm (Figure 2). After weaning, she had severe deficits, including right hemiplegia, facial palsy, expressive aphasia, slurred speech, and repetitive blinking. She was managed by a multidisciplinary team and treated with heparin (later switched to Clexane), dexamethasone, citicoline, levetiracetam, and early rehabilitation. At follow-up, she showed marked neurological recovery: near-normal gait, improved proximal right-arm movement (though with a weak grip), and improved speech. Tumor response remained good, supported by focal therapy (TTT), avoiding enucleation.

### 3.2. Systematic Review Results

#### 3.2.1. Study Selection

**Figure 3:**
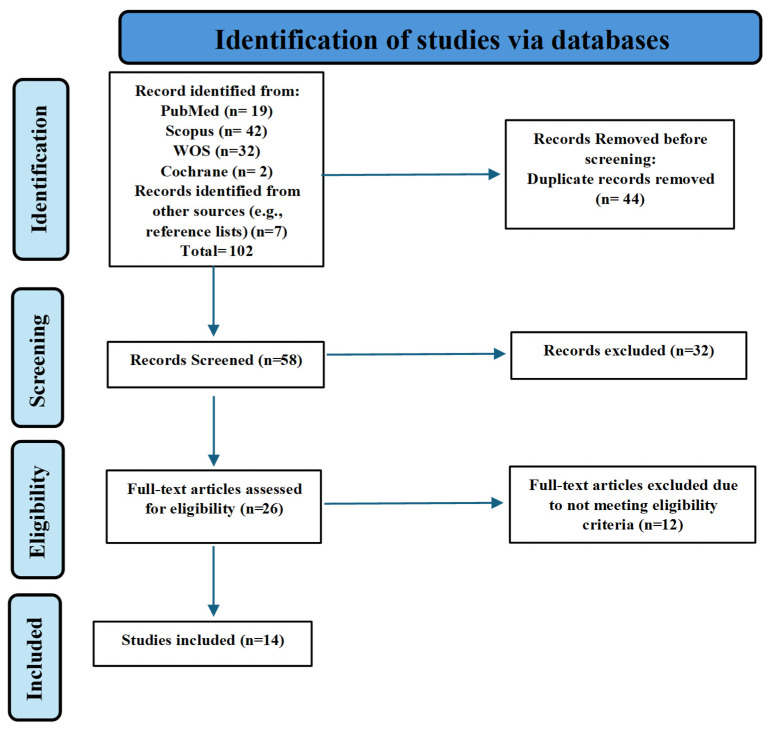
PRISMA flowchart.

A total of 102 records were identified through the literature search, including 19 from PubMed, 42 from Scopus, 32 from Web of Science, 2 from the Cochrane Library, and 7 additional records identified through reference list screening. After the removal of 44 duplicate records, 58 records remained and were screened based on titles and abstracts. Of these, 32 records were excluded for not meeting the inclusion criteria. The full texts of 26 articles were assessed for eligibility; 12 were excluded due to failure to meet the predefined eligibility criteria. As a result, 14 studies were included in the systematic review (Figure 3).

#### 3.2.2. Quality Assessment of Studies

The results of the quality assessment using the NOS and the JBI critical appraisal checklist are summarized in Table 2 and Table 3, respectively. According to the NOS, three studies achieved a maximum score of 9, one study scored 8, two studies scored 6, and one study scored 5. Three included cohort studies were single-arm studies; therefore, the comparability domain of the NOS was not applicable, and no stars were awarded for this domain.

Quality assessment using the JBI critical appraisal checklist indicated that among the seven included case series, three studies were of high quality; three were of moderate quality, primarily due to inadequate reporting of clear inclusion criteria; and one study was rated low quality.

#### 3.2.3. Studies and Overall Patient Characteristics

Overall, 11 (1.2%) CVA events were reported among 932 IAC-treated patients. The reported incidence of CVA varied across studies, ranging from 0% to 9.1% per patient, with higher proportions observed in small case series, and 0% to 2.2% per IAC procedure (Table 4). Given the heterogeneity in study design, sample size, and outcome reporting, these figures should be interpreted as descriptive estimates rather than pooled risk measures. (Note: Parks et al. [25] reported a case report of 2 cases of CVA without referral to the number of patients who received IAC, so this 100% risk per patient, and 20% per procedure, was not included in this estimation to avoid bias.) Among the 14 included studies, seven were cohort studies, and seven were case series. A total of 932 RB patients encompassing 1076 treated eyes underwent IAC, with study sample sizes ranging from two patients in a case series to 623 patients in a retrospective cohort study. Reporting of IAC exposure was heterogeneous across studies: some reported the cumulative number of IAC sessions, ranging from 10 to 2681 sessions, whereas others reported the mean number of sessions per patient, which ranged from 2.1 to 3.0. The age at IAC initiation varied widely, ranging from approximately 5 to 39 months across studies. Sex distribution was reported in most studies and was generally balanced between males and females in the overall IAC-treated population.

#### 3.2.4. Characteristics of RB Patients Who Developed CVA

Among patients who developed CVA, five had unilateral RB, while two patients were reported to have bilateral diseases. Four studies did not specify tumor laterality. Three patients experienced cerebrovascular events ipsilateral to the treated eye, whereas one patient developed a contralateral event in the left eye following treatment of the right eye. When available, ICRB classification ranged from Group C to Group E, although staging data were not reported in several cases (Table 5).

#### 3.2.5. IAC Characteristics and Clinical–Radiologic Features of CVA

Across the included studies, melphalan was the primary IAC agent used, either alone or in combination with carboplatin and/or topotecan. The number of IAC sessions before CVA varied widely, ranging from zero to seven sessions. All the procedures were performed via the femoral artery approach.

CVA occurred predominantly early after IAC or during the procedure itself. All reported events were ischemic in nature, including transient ischemic attacks (TIAs). The vascular territories involved were heterogeneous, affecting both anterior and posterior circulations, including the middle cerebral artery territory, basal ganglia, thalamus, occipital and parietal lobes, cerebellum (PICA), and basilar artery tip.

Clinical presentation ranged from asymptomatic radiologic findings to focal neurological deficits, seizures, hemiparesis, visual dysfunction, behavioral changes, and headache. Overall, no consistent pattern was identified regarding IAC regimen, number of sessions, vascular territory, or clinical presentation, as summarized in Table 6.

#### 3.2.6. Management, Outcomes, and Risk Factors of CVA

Management strategies for CVA varied across studies and ranged from conservative observation and supportive care to antiplatelet therapy, anticoagulation, intravenous methylprednisolone, and mechanical thrombectomy (Table 7). In multiple reports, acute management details were not specified.

Neurologic outcomes were favorable in nearly all cases, with full recovery reported in all patients at last follow-up. One study reported no recurrent infarcts after subsequent treatments. Radiologic evaluation was primarily performed using MRI, while CT, digital subtraction angiography (DSA), and MRI/MRA were also used in selected cases.

Continuation of IAC after CVA was possible in five patients, sometimes with protocol modification; however, several studies did not specify subsequent management. Follow-up duration, when reported, ranged from several months to multiple years.

Reported or suspected risk factors included thrombophilia (e.g., activated protein C resistance), difficult or prolonged ophthalmic artery catheterization, repetitive cannulation with possible vasospasm, suspected thromboembolic events, vascular dissection, atrial septal defect, younger age (<12 months), and potential carboplatin-related vascular toxicity. As a result, despite heterogeneous management approaches, neurologic prognosis was generally favorable.

## 4. Discussion

In this study, we showed that CVA is one of the complications that can happen during treatment for RB by IAC. To investigate this risk, we analyzed data from our institutional cohort alongside a meta-analysis that was replaced by a systematic review of the published literature. Within our small cohort, CVA was observed in 2.8% of procedures, affecting 9% of treated patients, while the systematic review identified CVA events in approximately 1.2% of reported cases overall. These findings suggest that CVA may be related to procedural, patient-specific, and treatment-related factors, particularly very young age at the time of intervention. Most affected patients experienced favorable outcomes; however, the potential for significant morbidity persists.

Marked variability exists in rates of reported complications after IAC for RB across regions and study designs, raising concerns about consistency and completeness of adverse-event reporting. For example, a United Kingdom series reported third cranial nerve palsies in ~17% of treated eyes [30]. In contrast, this complication has been infrequently reported in large North American cohorts [11,31], suggesting possible procedural differences or due to under-recognition and under-reporting. Similarly, metastatic rate has varied significantly in a systematic review of relatively small cohorts, ranging from 2.3 to 4.3% and up 25% [32], while it was less than 2% in different North American publications [11,31]. Regarding visual outcomes, a Japanese study reported visual acuity worse than 0.5 (20/40) in nearly half of treated eyes with normal macula [33], while European and North American studies show much lower rates of visual loss [11,30,31]. While these discrepancies may reflect heterogeneity in patient selection, disease stage, dosing, expertise, and follow-up, they may also be due to differences in reporting criteria, a limitation of the retrospective nature of these single-center studies. Systematic reviews note that many reports focus on the rate of eye globe salvage while incompletely documenting adverse events and lacking standardized definitions [34], potentially underestimating serious toxicities such as cerebrovascular accidents. This may explain the discrepancy between our cohort and the published literature, where we reported cerebrovascular accidents in 9% of patients (2.8% of procedures), exceeding the 1.2% rate in our systematic review. Underreporting, along with differences in cohort size, the reporting threshold for side effects, and patient- or procedure-specific factors, may contribute [35,36,37]. Patients who developed CVA in our cohort shared common features. All of them occurred ipsilateral to the treated eye, consistent with most reported cases and supporting a local vascular mechanism related to IAC (e.g., endothelial injury, vasospasm, thrombosis, or embolization) [38]. However, one case was reported where the contralateral size for the injection developed CVA [28], suggesting possible systemic embolic involvement and patient-related susceptibility. Of interest, CVA was reported in several patients with advanced ICRB groups D and E. This observation may reflect greater treatment intensity, including the need for multiple IAC sessions that may cause vascular damage due to cumulative vascular manipulation rather than tumor biology [12], although the available data do not allow determination of whether disease stage itself contributes to cerebrovascular risk. Events of CVA typically occurred during or shortly after the procedure, which may support a procedural contribution; however, the available evidence is insufficient to exclude the role of other patient- or treatment-related factors. Unfortunately, in our systematic review, we could not analyze the impact of the experience of the interventional radiologist on outcomes and rates of complications. On the other hand, IAC agents (melphalan, topotecan, carboplatin) may induce endothelial toxicity—via inflammation, oxidative stress, vasospasm, and thrombosis—especially when used in combination [39,40]. In previously reported cases, infarcts most frequently involved territories within the anterior circulation, including the basal ganglia, thalamus, corona radiata, parietal lobe, and the MCA territory, although posterior circulation involvement has also been described. Similarly, in our cohort, infarcts occurred within ICA territories, including the MCA, ACA, and the ICA itself. The occurrence of infarcts within these vascular territories, supplied by branches of the ICA, has clear anatomical continuity with the ophthalmic artery used for IAC delivery, supporting a potential procedure-related embolic or vasospastic mechanism [41]. Clinically, the presentation of CVA in both our cohort and the cases reported in the literature ranged from asymptomatic radiologic findings to overt neurological manifestations, including focal seizures, hemiparesis, slurred speech, behavioral changes, visual dysfunction, and severe headache. The presence of a proportion of asymptomatic or minimally symptomatic cases suggests that many CVA events may be subclinical and detectable only on neuroimaging. Additionally, instances of clinically suspected events without corresponding radiologic abnormalities (TIA) further suggest that some cases may go undetected, contributing further to potential underreporting of CVA incidence. This variability indicates the challenges of recognizing pediatric strokes [42,43] and highlights the importance of maintaining a high index of suspicion and maybe considering post-IAC neuroimaging in order not to miss such cases. The imaging modalities that were mainly used included CT, MRI, and cerebral angiogram, depending on clinical emergency and variability. MRI is generally considered the preferred imaging modality for pediatric stroke, as it provides greater sensitivity for detecting early ischemic changes and small infarcts that may not be visible on CT [44].

Management of CVA varied widely in our cohort and the reported literature, reflecting the lack of standardized protocols for IAC-related stroke [45], due to the rarity of this complication and the absence of evidence-based guidelines. Neurological outcomes were generally favorable, with most patients achieving full recovery. In our cohort, one patient with severe initial deficits improved markedly, regaining near-normal gait and partial upper limb function, with only mild residual deficits. Favorable outcomes likely reflect early recognition, prompt imaging, and supportive care, as well as the potential reversibility of procedure-related events such as vasospasm or transient ischemia. Despite this, CVA remains clinically significant, requiring close monitoring for early detection. Long-term follow-up is essential, as pediatric stroke survivors may develop delayed neurological or cognitive sequelae, emphasizing the need for ongoing multidisciplinary care [46].

The occurrence of CVA presents a major ethical and clinical dilemma regarding whether to continue IAC or discontinue treatment, with possible need for enucleation. In some cases, IAC was stopped to avoid another possible CVA and prevent permanent neurological deficits, including one in our cohort. In others, treatment continued with modifications (e.g., dose adjustment or changing agents), or patients were shifted to alternative RB therapies. These decisions reflect the need for balance between maintaining oncologic control and minimizing serious neurological risk. Clinicians often consider potential individual risk factors and may avoid repeating the same intervention to reduce the likelihood of such a serious complication.

Findings from our cohort and systematic review suggest that CVA after IAC for RB is multifactorial. Several factors have been proposed as possible contributors, including young age, small vessel caliber, prothrombotic conditions, advanced disease requiring multiple treatment sessions, repeated catheterization, vasospasm, vascular injury, embolization, and potential drug-related endothelial toxicity. However, these observations are derived from a limited number of events reported across heterogeneous studies, many of which lacked detailed procedural and comparative data. Therefore, these factors should be regarded as hypotheses-generating observations rather than established risk factors. Young age and small vessel caliber may increase vulnerability to vascular injury, while prothrombotic tendencies can predispose to thromboembolic events. Advanced disease and bilateral RB often require repeated sessions, increasing cumulative vascular trauma. Procedural factors—such as repeated catheterization, vasospasm, prolonged duration, and embolization—also contribute, alongside drug-related endothelial toxicity and inflammation [17,20,22,24,28]. Recognizing these mechanisms is essential to reducing risk. Preventive strategies include meticulous catheterization by experienced operators, minimizing procedure time and manipulation, and close neurological monitoring with early imaging when deficits are suspected. These measures may improve IAC safety while preserving its therapeutic benefit. Despite being the first study to specifically evaluate CVA events and explore potential risk factors in RB patients undergoing IAC, several limitations should be acknowledged. First, the cohort size was relatively small. In addition, the systematic review was limited by incomplete reporting in the existing literature, as many published cases lacked detailed descriptions of CVA characteristics, procedural variables, and patient clinical features. This limited reporting restricted the ability to perform more comprehensive risk factor analysis or meta-analysis.

In conclusion, this study provides a comprehensive assessment of CVA in RB patients treated with IAC, combining institutional and systematic review data. Although uncommon, CVA is a serious and potentially underrecognized complication, particularly in very young patients. The higher incidence in our cohort suggests possible underreporting and variability in detection. The predominance of ipsilateral events, procedural timing, and ICA involvement suggests mainly a procedure-related mechanism influenced by patient susceptibility and treatment intensity. Outcomes were generally favorable with early recognition and supportive care, though significant morbidity remains possible. These findings highlight the need for careful procedure techniques, close attention from clinicians, and regular monitoring of neurological status. More multicenter studies with standardized reporting are needed to better identify risk factors and improve the safety of IAC.

## Figures and Tables

**Table 1:** Baseline characteristics and outcomes of the cohort (*n* = 33).

Feature		Number (%)	Number of Patients with Stroke (%)	*p*-Value
Number of patients		33	3 (9.1%)	-
Number of procedures		104	3 (2.9%)	-
Age at diagnosis (Months) Mean (Range)		19.1 (1–72)	-	-
Age at 1st injection	>1 Year	7 (21.2%)	2 (28.6%)	0.11
	≥1 Year	26 (78.8%)	1 (3.8%)	
Gender	Male	12 (36.4%)	1 (8.3%)	1.0
	Females	21 (63.6%)	2 (9.5%)	
Laterality	Unilateral	16 (48.5%)	1 (6.3%)	1.0
	Bilateral	17 (51.5%)	2 (11.8%)	
ICRB stage	C	7 (21.2%)	0	0.35
	D	23 (69.7%)	2 (8.7%)	
	E	2 (6.1%)	1 (50%)	
Indication	Primary	12 (36.4%)	1 (8.3%)	1.0
	Secondary	21 (63.6%)	2 (9.5%)	
Melphalan dose	20 Microgram	7 (21.2%)	2 (28.6%)	0.16
	25 Microgram	21 (63.6%)	1 (4.8%)	
	30 Microgram	5 (15.2%)	0	
Number of IAC injections Median (Range)		3 (1–6)	-	-
Site of injection	Right	17 (51.5%)	2 (11.8%)	1.0
	Left	16 (48.5%)	1 (6.3%)	
CVA occurrence		3 (9.1%)	-	-
Enucleation		13 (39.4%)	-	-
Survival		31 (93.9%)	-	-

**Table 2:** Quality assessment of cohort studies.

Reference	Selection	Comparability	Outcomes	Quality Score
Schreiber et al., 2026 [17]	***	**	***	8
Requejo et al., 2018 [18]	***	NA	**	5
Munier et al., 2011 [19]	***	NA	***	6
Batu Oto et al., 2020 [20]	****	**	***	9
Ammanuel et al., 2018 [21]	****	**	****	9
Leal-Leal et al., 2016 [22]	***	NA	***	6
Munier et al., 2017 [23]	****	**	***	9

**Note:** NA = not applicable. The quality assessment was performed using the Newcastle–Ottawa Scale (NOS). Stars indicate the number of points awarded within each domain (** = 2 points; *** = 3 points; **** = 4 points).

**Table 3:** Quality assessment of case series studies.

References	Q1	Q2	Q3	Q4	Q5	Q6	Q7	Q8	Q9	Q10	Quality Rating
Wai et al., 2023 [10]	No	Yes	Yes	Yes	Yes	Yes	No	Yes	Yes	Yes	Moderate
Rojanaporn et al., 2019 [24]	No	Yes	Yes	Yes	Yes	Yes	No	Yes	Yes	Yes	Moderate
Parks et al., 2025 [25]	No	Yes	Yes	Yes	Yes	Yes	Yes	Yes	No	NA	Moderate
Shields et al., 2015 [26]	Yes	Yes	Yes	Yes	Yes	Yes	Yes	Yes	Yes	Yes	High
Shields et al., 2014 [27]	Yes	Yes	Yes	Yes	Yes	Yes	Yes	Yes	Yes	Yes	High
Mengüşoğlu et al., 2025 [28]	No	Yes	Yes	No	No	Yes	Yes	Yes	No	NA	Low
Ghassemi et al., 2014 [29]	Yes	Yes	Yes	Yes	Yes	No	Yes	Yes	Yes	Yes	High

**Table 4:** Overall IAC population.

Author and Year	Country	Study Design	Total Number of RB Patients Treated with IAC (No. of Eyes)	Total Number of IAC Sessions	Age at IAC (Overall Population)	Gender (M/F, Overall Population)	Number of CVA Events (per Patient, %)	Number of CVA Events (per Procedure, %)
Schreiber et al., 2026 [17]	USA	Cohort	623 patients (784)	2681	Mean: 18.9 months	312/311	2 (0.3%)	0.07%
Wai et al., 2023 [10]	Malaysia	Case series	20 patients (22)	46	Mean: 21.3 months	12/8	1 (5%)	2.2%
Rojanaporn et al., 2019 [24]	Thailand	Case series	26 patients (27)	73	Mean: 23 months	13/13	1 (3.8%)	1.4%
Requejo et al., 2018 [18]	Argentina	Cohort	41 patients (45)	248	Mean: 19.2 months	21/20	ZERO	NA
Parks et al., 2025 [25]	USA	Case series	2 patients (3)	10	1st patient: 14 months 2nd patient: 10 months	Both females	2 (100%)	20%
Munier et al., 2011 [19]	Switzerland	Cohort	13 patients (13)	NR	Mean: 26 months	NR	ZERO	NA
Batu Oto et al., 2020 [20]	Turkey	Cohort	30 patients (31)	NR	*	NR	1 (3.3%)	NA
Shields et al., 2015 [26]	USA	Case series	12 patients (12)	70 (Initial + rescue)	NR	4/8	ZERO	NA
Shields et al., 2014 [27]	USA	Case series	67 patients (70)	Mean: 3	Mean: 30 months	26/41	ZERO	NA
Mengüşoğlu et al., 2025 [28]	Switzerland	Case series	2 patients (2)	NR	1st patient: 5 months 2nd patient: 11 months	NR	2 (100%)	NA
Ammanuel et al., 2018 [21]	USA	Cohort	36 patients (43)	125	Mean: 15.1 months	63/62	1 (2.8%)	0.8%
Leal-Leal et al., 2016 [22]	Mexico	Cohort	11 patients (11)	NR	NR	7/4	1 (9.1%)	NA
Munier et al., 2017 [23]	Switzerland	Cohort	25 patients (25)	2.7 ± 0.5	Mean: 33.3 months	NR	ZERO	NA
Ghassemi et al., 2014 [29]	Iran	Case series	24 patients (24)	33	Mean: 38.9 months	NR	ZERO	NA
Overall			932 patients (1076)				11 (1.2%)	

* Age distribution (months) (group 1/group 2): 0–6 m: (1/3), 6–12 m: (7/4), 12–36 m: (9/2) >36 m: (4/0). NR: Not reported, NA: Not Applicable.

**Table 5:** Baseline characteristics of RB patients who developed CVA.

Author and Year	Number of CVA Cases	Laterality of RB	Eye Affected by CVA Relative to RB (Ipsilateral/Contralateral/Bilateral)	ICRB Group
Schreiber et al., 2026 [17]	2	Unilateral for both cases	NR	NR
Wai et al., 2023 [10]	1	NR	NR	E
Rojanaporn et al., 2019 [24]	1	NR	NR	NR
Parks et al., 2025 [25]	2	1st patient: Bilateral 2nd patient: Unilateral (Right eye)	1st patient: bilateral 2nd patient: ipsilateral	1st patient: RT: E, LT: D 2nd patient: C
Batu Oto et al., 2020 [20]	1	Unilateral	NR	NR
Mengüşoğlu et al., 2025 [28]	2	1st patient: Unilateral Right eye RB 2nd patient: Unilateral Left eye RB	1st patient: contralateral 2nd patient: ipsilateral	1st patient: C 2nd patient: D
Ammanuel et al., 2018 [21]	1	NR	NR	NR
Leal-Leal et al., 2016 [22]	1	Bilateral	Ipsilateral to the treated eye (left)	C

**Table 6:** IAC Procedural characteristics and CVA features.

Author and Year	IAC Agent Used **	Number of IAC Sessions Prior to CVA	Timing of CVA	Type of CVA	Vascular Territory Involved	Clinical Presentation
Schreiber et al., 2026 [17]	Melphalan ± carboplatin ± topotecan.	1st patient: 2 sessions 2nd patient: 1 session	1st patient: Early 2nd patient: Early	Ischemic	1st patient: Right thalamus and basal ganglia 2nd patient: Right corona radiata	Asymptomatic
Wai et al., 2023 [10]	-Melphalan alone (1 eye) -Melphalan + Topotecan (21 eyes)	NR	Early	Ischemic	Right MCA	Left-sided focal seizure post IAC
Rojanaporn et al., 2019 [24]	Melphalan and carboplatin	7 sessions	Early	Ischemic (TIA)	NR	NR
Parks et al., 2025 [25]	Combination of topotecan, melphalan, and carboplatin	1st patient: 4 sessions 2nd patient: 6 sessions	Early	1st patient: ischemic 2nd patient: ischemic (TIA)	1st patient: Left occipital lobe (cortical/subcortical) 2nd patient: Right middle frontal gyrus (MCA/ACA); right optic nerve/chiasm	1st patient * 2nd patient **
Batu Oto et al., 2020 [20]	-Melphalan alone -Melphalan + Topotecan	NR	Early	Ischemic	NR	NR
Mengüşoğlu et al., 2025 [28]	NR	1st patient: 1 session 2nd patient: Zero	During the procedure	Ischemic	1st patient: Posterior circulation—cerebellar (left PICA) 2nd patient: Posterior circulation—basilar artery tip	For both pts: Asymptomatic
Ammanuel et al., 2018 [21]	Melphalan ± carboplatin ± topotecan	NR	Early	Ischemic	Internal carotid artery territory (anterior circulation)	Seizure
Leal-Leal et al., 2016 [22]	Melphalan + Topotecan	Zero	During the procedure	Ischemic	Left parietal lobe	Hemiparesis and severe ipsilateral headache

* Focal neurological deficit (behavioral change, staring at objects for long periods, inability to track or reach for objects, visual dysfunction). ** Asymptomatic (abnormal tongue movement).

**Table 7:** Management, outcomes, and reported risk factors of CVA.

Author and Year	Acute CVA Management	Neurologic Outcome at Last Follow-Up	Radiologic Method Used	Continuation of IAC After CVA	Follow-Up Duration	Reported Risk Factors for CVA
Schreiber et al., 2026 [17]	NR	No recurrent infarcts reported after subsequent treatments *	MRI	IAC continued	NR (follow-up schedule described; duration not specified)	1st patient: Activated protein C resistance (thrombophilia). 2nd patient: Difficult OA catheterization; multiple catheterization attempts.
Wai et al., 2023 [10]	NR	Full recovery	CT	NR	Mean: 30.7 months	NR
Rojanaporn et al., 2019 [24]	Intravenous methylprednisolone	Full recovery	NR	Continued with protocol modification (carboplatin discontinued).	Mean: 32 months	Suspected vasospasm from repetitive IAC cannulation; possible carboplatin-related vascular toxicity
Parks et al., 2025 [25]	Conservative with observation and supportive care only.	Full recovery	1st patient: MRI: focal T2/FLAIR. 2nd patient: MRI: diffusion-weighted *	NR	NR	NR
Batu Oto et al., 2020 [20]	Antiplatelet therapy	Full recovery	NR	NR	Median: 40.8 months	-Suspected thrombosis or vascular dissection -Atrial septal defect (possible paradoxical embolism) -Prolonged OA catheterization time
Mengüşoğlu et al., 2025 [28]	*	For both patients: Full neurological recovery	For both: DSA (acute), MRI/MRA (follow-up)	For both pts: IAC continued (eye salvage implied)	6 months	Thromboembolic (suspected)
Ammanuel et al., 2018 [21]	NR	Full recovery	MRI	NR	Mean: 954 days	NR
Leal-Leal et al., 2016 [22]	NR	Full recovery	MRI	IAC continued	122 days	Prolonged OA catheterization (~30 min); suspected vasospasm

* 1st patient: Antiplatelet + anticoagulation, mechanical thrombectomy (aspiration). 2nd patient: Antiplatelet therapy, mechanical thrombectomy (aspiration).

## Data Availability

The research data are available upon reasonable request.
